# Apical Localization of Zinc Transporter ZnT4 in Human Airway Epithelial Cells and Its Loss in a Murine Model of Allergic Airway Inflammation

**DOI:** 10.3390/nu3110910

**Published:** 2011-10-25

**Authors:** Chiara Murgia, Dion Grosser, Ai Q. Truong-Tran, Eugene Roscioli, Agnes Michalczyk, Margaret Leigh Ackland, Meredin Stoltenberg, Gorm Danscher, Carol Lang, Darryl Knight, Giuditta Perozzi, Richard E. Ruffin, Peter Zalewski

**Affiliations:** 1 INRAN-National Research Institute on Food &amp; Nutrition, Roma 00178, Italy; Email: murgia@inran.it (C.M.); perozzi@inran.it (G.P.); 2 Discipline of Medicine, University of Adelaide, The Queen Elizabeth Hospital, Woodville, South Australia 5011, Australia; Email: dion.grosser@health.sa.gov.au (D.G.); ai.truongtran@gmail.com (A.Q.T.-T.); eugene.roscioli@adelaide.edu.au (E.R.); carol.lang@adelaide.edu.au (C.L.); carol.lang@adelaide.edu.au (R.E.R.); 3 Centre for Cellular and Molecular Biology, School of Biological and Chemical Sciences, Deakin University, Burwood, Victoria 3125, Australia; Email: agnesm@deakin.edu.au (A.M.); leigh.ackland@deakin.edu.au (M.L.A.); 4 Institute of Biomedicine, Neurobiology, Aarhus University, DK-8000 Aarhus C, Denmark; Email: meredinstoltenberg@hotmail.com (M.S.); gd@neuro.au.dk (G.D.); 5 James Hogg iCAPTURE Centre for Cardiovascular and Pulmonary Research, St Paul’s Hospital, 1081 Burrard Street, Vancouver, BC, V6Z 1Y6, Canada; Email: dknight@mrl.ubc.ca

**Keywords:** zinc, zinc transporter, ZnT4, airway epithelium, airway inflammation, asthma, Zinquin, Se-Autometallography (Se-AMG)

## Abstract

The apical cytoplasm of airway epithelium (AE) contains abundant labile zinc (Zn) ions that are involved in the protection of AE from oxidants and inhaled noxious substances. A major question is how dietary Zn traffics to this compartment. In rat airways, *in vivo* selenite autometallographic (Se-AMG)-electron microscopy revealed labile Zn-selenium nanocrystals in structures resembling secretory vesicles in the apical cytoplasm. This observation was consistent with the starry-sky Zinquin fluorescence staining of labile Zn ions confined to the same region. The vesicular Zn transporter ZnT4 was likewise prominent in both the apical and basal parts of the epithelium both in rodent and human AE, although the apical pools were more obvious. Expression of ZnT4 mRNA was unaffected by changes in the extracellular Zn concentration. However, levels increased 3-fold during growth of cells in air liquid interface cultures and decreased sharply in the presence of retinoic acid. When comparing nasal *versus* bronchial human AE cells, there were significant positive correlations between levels of ZnT4 from the same subject, suggesting that nasal brushings may allow monitoring of airway Zn transporter expression. Finally, there were marked losses of both basally-located ZnT4 protein and labile Zn in the bronchial epithelium of mice with allergic airway inflammation. This study is the first to describe co-localization of zinc vesicles with the specific zinc transporter ZnT4 in airway epithelium and loss of ZnT4 protein in inflamed airways. Direct evidence that ZnT4 regulates Zn levels in the epithelium still needs to be provided. We speculate that ZnT4 is an important regulator of zinc ion accumulation in secretory apical vesicles and that the loss of labile Zn and ZnT4 in airway inflammation contributes to AE vulnerability in diseases such as asthma.

## 1. Introduction

Airway epithelium (AE) is a pseudo-stratified columnar epithelium that lines the conducting nasal and tracheo-bronchial airways of the respiratory system. Amongst many things, AE provides a physical barrier to inhaled noxious substances, produces smooth muscle relaxing factor and provides mucociliary clearance that helps to maintain airway sterility and integrity [1,2]. The predominant cell type in AE is the ciliated columnar cell, each containing a few hundred cilia which protrude into the airway lumen and beat in a coordinated manner to force mucin and trapped foreign substances up the respiratory tract. AE is altered and often damaged by chronic smoking and in chronic inflammatory airway diseases such as asthma [3,4].

One factor that may influence airway integrity is the essential micronutrient zinc (Zn), a metal belonging to the group IIb series. Zn is the second most abundant metal in the body although approximately two-fold less than iron. The metal is obtained largely from protein-rich foods. Zn is involved in many physiological and pathological processes being present in a multitude of metalloenzymes and an essential player in the three dimensional structure of many proteins; free or labile Zn ions play important roles as anti-oxidants and in the regulation of cell death pathways [5]. Of particular importance is the central role it plays in the immune system, in the central nervous system and in epithelial integrity [6].

Zinc deficiency was reported to complicate some chronic diseases, including asthma [7]. The NHANES II survey of 9074 adults in the general population in USA found a negative relationship between wheezing and the serum Zn to Cu ratio [8] while a case-control study in Scotland noted an increase in the presence of atopy, bronchial reactivity and the risk of allergic-type symptoms in adults with the lowest intake of dietary Zn [9]. Several other studies (reviewed in [10]) have revealed that asthmatics are likely to have hypozincaemia and/or low hair Zn levels, suggestive of an underlying Zn deficiency or, at least, altered Zn metabolism. Particularly affected by Zn deficiency is the AE, which is normally quite resistant to oxy-radical and cytokine-induced apoptotic cell death but becomes susceptible to damage when depleted of Zn *in vitro* [11,12]. *In vivo*, mild nutritional Zn deprivation significantly worsened airway inflammation in the mouse allergic airway inflammation model and this was associated with high rates of apoptosis of epithelial cells in AE [13]. The major bulk of tissue Zn is tightly incorporated in metalloenzymes and Zn finger transcription factors. Other pools of loosely bound (labile) Zn are involved in growth regulation, apoptosis, cell signaling and secretion (reviewed in [14,15]). These labile pools have been visualized using Zn-specific fluorophores including Zinquin [16] or by conversion *in vivo* and *in vitro* to Zn-sulphur or Zn-selenium nanocrystals, which are then made visible in semi-thin Epon sections by autometallographic (AMG) silver enhancement [17,18,19,20]. 

In our previous studies, when Zinquin was added to cryosections of trachea or lung, strong fluorescence was seen at the luminal end of the airway epithelial cells, suggesting an abundance of labile Zn in the apical cytoplasm of the columnar cells. This was confirmed by adding Zinquin to isolated normal human AE cells (hAEC); strong fluorescence was observed in their apical cytoplasm, immediately below the ciliary apparatus and within the cilia, themselves [12,14,21]. Delivery of Zn ion to the apical AE cytoplasm may involve a vesicular pathway since the Zinquin studies showed a punctate fluorescence indicative of pools of labile Zn within some form of cytoplasmic vesicle (zincosome), in accordance with what has been shown in other cell types [22].

Intracellular zinc homeostasis is achieved by the activity of specific proteins involved in uptake, efflux and intracellular compartmentalization. In the past decade a number of mammalian zinc transporters have been identified and the corresponding genes cloned [7,23,24]. Based on their sequence homology and structural properties, they have been assigned to two distinct families: SLC39A (or ZIP, ZRT/IRT-related protein) and SLC30A (or ZnT). ZIPs are mainly involved in uptake of Zn across the plasma membrane into cytosol; while ZnTs are believed to facilitate efflux of Zn from cells and mobilization of the metal in intracellular organelles [24]. It is believed that Zn ions, derived from a circulating reservoir of Zn ion carrier proteins in the sub-epithelial capillaries, are transported into the AE by one of the plasma membrane Zn transporters. Gene expression analysis of Zn transporters have been carried out in several tissues showing that any given cell type expresses a set of these proteins [24]. With respect to airway Zn, knowledge has mostly derived from studies in animal models. A comprehensive gene expression analysis of Zn transporters in the lungs of Balb/c mice by real time qPCR revealed expression of several genes belonging to both ZIP and ZnT families with a prevalence of ZIP1, ZIP6, ZnT1, ZnT4 and ZnT6 [25]. Our previous study has shown the importance of Zn ions in the maintenance of airways homeostasis in the ovalbumin (OVA) induced acute, allergic airway inflammation model [13]. In this model that replicates some of the features of human asthma, these mice show marked reductions in the labile apical Zn pools in AE [13]. In this model the effect of inflammation on the expression of Zn transporters was up-regulation, (>2-fold) of ZIP1, ZIP6 and ZIP14 and down-regulation (>2-fold) in ZIP2, ZIP4, ZIP7, ZnT6 and ZnT4, with the latter showing the most pronounced decrease [25].

The vesicular Zn transporter ZnT4 [22,26,27] is thought to be responsible for packaging Zn in cytoplasmic vesicles and endosomes. The gene was initially identified as the molecular basis of the mouse mutant *lethal milk* syndrome in which the ZnT4-*lm* transcript carries a premature stop codon at amino acid 297 and the incomplete protein is then rapidly degraded [28]. Dams carrying this mutation produced milk with reduced content of Zn, pointing to ZnT4 as important for Zn secretion [26,29]. ZnT4 belongs to a subfamily of ZnT transporters, which includes also ZnT2 and ZnT8. These proteins share higher degree of homology among themselves than with the remaining ZnT family members and they all appear to associate with specific secretory vesicles [30,31,32].

The substantial down regulation of ZnT4 mRNA expression in the mice with airway inflammation [25] suggests that this transporter may also be of relevance to changes in Zn metabolism in airway inflammatory diseases such as asthma. This study was limited, however, by the lack of knowledge as to the level of expression of ZnT4 in different lung cell types and whether this was reflected at the protein level. The major aim of the current study was to investigate the subcellular distribution of ZnT4 protein in human AE as well as in murine AE, before and after induction of allergic airway inflammation. In addition, we used Se-AMG to further explore intracellular Zn localization and distribution in AE.

## 2. Materials and Methods

### 2.1. Airway Epithelial Cells and Cultures

Normal human bronchial/tracheal epithelial cells (NHBE, passage 1) and Bronchial Cell Epithelial Growth Media (BEGM) were purchased from Lonza Australia, Mt Waverley. Cells were expanded (2 passages) and stored in aliquots in liquid nitrogen according to manufacturer’s instructions. For NHBE cultures, cells were seeded onto filters to near-confluent levels on 0.4 mm PET transparent transwell filters (Becton-Dickinson, France) in BEGM medium. Filters were maintained submerged until day 7 and then the medium was removed from the apical side and continued as air liquid interface cultures. On day 9, some filters received all-trans retinoic acid (RA, 50 nM, Sigma Chemicals, St Louis, MO, USA) in the basal medium. RA was replenished daily. 

Human nasal brushings were obtained using a cytology brush from consenting volunteers. Human bronchial brushings were collected from consenting patients undergoing bronchial biopsies at The Queen Elizabeth Hospital. Samples were taken from healthy looking tissues lining the bronchi through a vigorous brushing action and placed in BEGM medium. Cells were cytospun onto slides or used immediately as cell suspensions for further studies. In some subjects, both nasal and bronchial cells were collected for comparison. All procedures were approved by The Human Ethics Committee of The Queen Elizabeth Hospital. 

### 2.2. Measurement of Trans-Epithelial Electrical Resistance (TEER)

TEER of NHBE cells in air liquid interface cultures was measured daily using a volt ohmmeter (EVOM, WPI, Sarasota, FL, USA) according to manufacturer’s instructions. Before measurement, medium was added to apical side of wells and allowed to equilibrate for 10 min.

### 2.3. Quantitative Real Time RT-PCR (qRT-PCR)

After collection, airway epithelial cells (AEC) were immediately placed in RLT buffer and total RNA was isolated and digested with DNase, as per kit instructions (RNeasy mini kit, Qiagen, Doncaster, Victoria, Australia). cDNA was prepared from 2 µg of total RNA using a High capacity cDNA reverse transcription kit (Applied Biosystems, Mulgrave Victoria, Australia). PCR was performed using 50 µg of cDNA as template and pre-made TaqMan primer/probes (SLC39A3 Hs00536788_m1, SLC39A4 Hs00214912_m1, SLC39A6 Hs00202392_m1, SLC39A7 Hs00199596_m1, SLC39A8 Hs00223357_m1, SLC39A14 Hs00299262_m1, SLC30A4 Hs00203308_m1, SLC30A6 Hs00215827_m1. Primer/probes, TaqMan gene expression master mix and the 7300 Real Time PCR system were all from Applied Biosystems (Mulgrave Victoria, Australia). PCR conditions were standard; 50 °C for 2 min, 95 °C for 10 min, (95 °C for 15 s, 60 °C 1 min), 40 cycles. Hypoxanthine guanine phosphoribosyl transferase (HPRT) or glyceraldehyde 3-phosphate dehydrogenase (GAPDH) house-keeping genes were used as the internal standards. A sample without reverse transcriptase enzyme was included to check for DNA contamination. The cycle thresholds (*C*_t_) were obtained for test and house-keeping genes, and their differences calculated (Δ*C*_t_). Results were presented as log*_2_ RQ* (relative quantity) as fold difference compared to housekeeping gene (1^−2Δ*C*t^) as described by Applied Biosystems (User Bulletin #2ABI).

### 2.4. Zinquin Labeling

Zinquin, ethyl-[2-methyl-8-p-toluenesulphonamido-6-quinolyloxy]acetate (Dr. A.D. Ward, Department of Chemistry, University of Adelaide, South Australia) was dissolved in dimethylsulfoxide (DMSO) at 5 mM and stored at −20 °C in the dark. Cells were incubated for 30 min in 25 µM Zinquin at room temperature (RT). Zinquin was freshly diluted in 1× phosphate-buffered saline (PBS) immediately before addition. A Bio-Rad MRC-1000 UV Laser Scanning Confocal Microscope System, equipped with UV-Argon laser, was used in combination with a Nikon Diaphot 300 inverted microscope in fluorescence mode [21]. Fluorescence excitation was at 351/8 nm and emission at 460 long pass (LP). Images were collected using a 40× water immersion objective lens with NA 1.15. Each image was averaged over 6 scans by Kalman filtering. 

### 2.5. Immunofluorescence and Antibodies

Air dried cryosections and cytospun cells were fixed in 100% chilled acetone for 10 min at RT while cytospun cells were fixed in 4% paraformaldehyde for 15 min at RT, before washing with PBS containing 4% BSA (3× for 5 min each), to minimize non-specific binding. Primary antibody used in this study was rabbit polyclonal anti-ZnT4 [22]. Rabbit anti-rat ZnT4 was used at 1:250 dilution overnight at RT in a humidified chamber. Secondary antibody was fluoroscein isothiocyanate-conjugated goat anti-rabbit IgG (Rocklands, Gilbertsville, PA, USA) at 1:250 dilution for 2 h at RT. A drop of Anti-Fade fluorescent mounting medium (DAKO Corporations, CA, USA) was added, the coverslip-mounted and immediately (Excitation 488/10 nm and emission at 522/35).

### 2.6. Dual Labeling of ZnT4 and Zinc

For dual labeling studies of ZnT4 and Zn on cytospun cells, a similar protocol was used for that of ZnT4 alone except that, after secondary antibody addition, cells were labeled for Zn by Zinquin. Slides were washed and Zinquin was added to a final concentration of 25 μM in PBS. Slides were washed again in PBS and mounted as above. Where dual staining was performed, fluorescence images were merged using Confocal Assistant (Version 4.02) software package.

### 2.7. Murine Airway Inflammation Model

All experiments were performed under the University of Adelaide Animal Ethics Committee, approval number M-54-2001 and in compliance with “Principles of Animal Care” publication number 86-23 of the National Institute of Health and the “Australian Code of Practice for the Care and Use of Animals for Scientific Purposes”, 6th ed. The method is described in detail elsewhere [13]. Briefly, female Balb/c mice (age 4-6 weeks; pathogen free) were randomly divided into experimental groups and housed at 21 °C with a 14-h light/10-h dark cycle. Mice received 50 μg of chicken ovalbumin (OVA) per 1 mL of alhydrogel (CSL, Parkville, Australia) in 0.9% sterile saline, intra-peritoneal (i.p.) on days 0 and 14. Control (SAL) mice received alhydrogel in 0.9% saline alone. Mice sensitized to OVA were then aerochallenged with 10 mg/mL OVA in 0.9% saline from day 22 to day 32, for 30 min, three times a day, every 2nd or 3rd day, using a side-stream nebulizer, which produced particles of 1-3 μm (Fisher and Paykel, Sydney, Australia). SAL-treated groups were nebulised with 0.9% saline alone. Mice were sacrificed over 2 days, on the day following their last nebulisation. Tissues were collected and processed as described in [13]. 

Cryosections of ~5 μm width were cut from regions of the lung rich in airways. Images (both bright-field and fluorescence) were captured at several sites along the epithelium. Profile lines were drawn across the epithelium based on bright-field photographs and superimposed on the corresponding fluorescence images. This was necessary for the OVA-treated mice as it was often difficult to see the epithelium in the fluorescence sections. At least 5 lines were drawn for each image for 5-10 images at randomly-chosen spots along the AE for each mouse); a total of 10 SAL and 11 OVA mice were measured in this way. Because of varying epithelial thickness, mean fluorescence intensity was calculated for each tenth interval across the epithelium, beginning at the luminal surface and ending at the basal surface. 

### 2.8. *In Vitro* Se Autometallography in Pig Tracheal Epithelial Cells

Pig trachea was obtained freshly from the local abattoir. Ciliated epithelial cells were dislodged by rapid shaking, washed and resuspended to 10^7^/mL in 5 mL of 0.1M PBS (Sorenson’s, pH 7.4) containing 0 or 10 mg/mL sodium selenide (Sigma). After 60 min at RT, 5 mL of 3% glutaraldehyde in PBS was added and cells fixed overnight at RT. The following day cells were washed 3× with PBS. Cells were smeared on glass slides, air-dried and stained with AMG developer [17,18] for 70 min at 26 °C in a dark box. Smears were post-fixed with 70% ethanol for 30 min and counterstained with 0.1% aqueous toluidine blue pH 4.0. Zn staining was assessed by light microscopy. 

### 2.9. *In Vivo* Se Autometallography in Rat Tracheal Epithelium

The specificity of this technique for detecting Zn ions has previously been well-characterized in sections of brain and other tissues [18]. Zn-Se AMG has not previously been used for tracing of Zn ions in lung tissue and minor adaptations have therefore been introduced in order to obtain maximum outcome. Male Wistar rats were euthanized and 10 mL of a 10 mg/mL solution of sodium selenide was infused into the trachea and lungs for 3 min, followed by 3% glutaraldehyde in PBS for a further 3 min. Lungs and trachea were removed, cut into 4 pieces each and left in 3% glutaraldehyde in PBS overnight at RT. 100 μm sections were cut using a vibratome, dipped in 0.5% gelatin and developed with AMG while floating, according to standard methods as described elsewhere [17,18]. To stop the reaction, 5% sodium thiosulphate was added for 10 min. Sections were rinsed in distilled water, counterstained with 0.1% toluidine blue and examined by light microscopy. Interesting areas were cut into smaller blocks, fixed in 1% osmium tetroxide for 1 h and stained with uranyl acetate. Sections were alcohol-fixed, embedded in Epon and prepared for transmission electron microscopy. 

## 3. Results

### 3.1. Zinquin Fluorescence and Autometallography of Metal Ions in Apical Cytoplasm of AE

**Figure 1:**
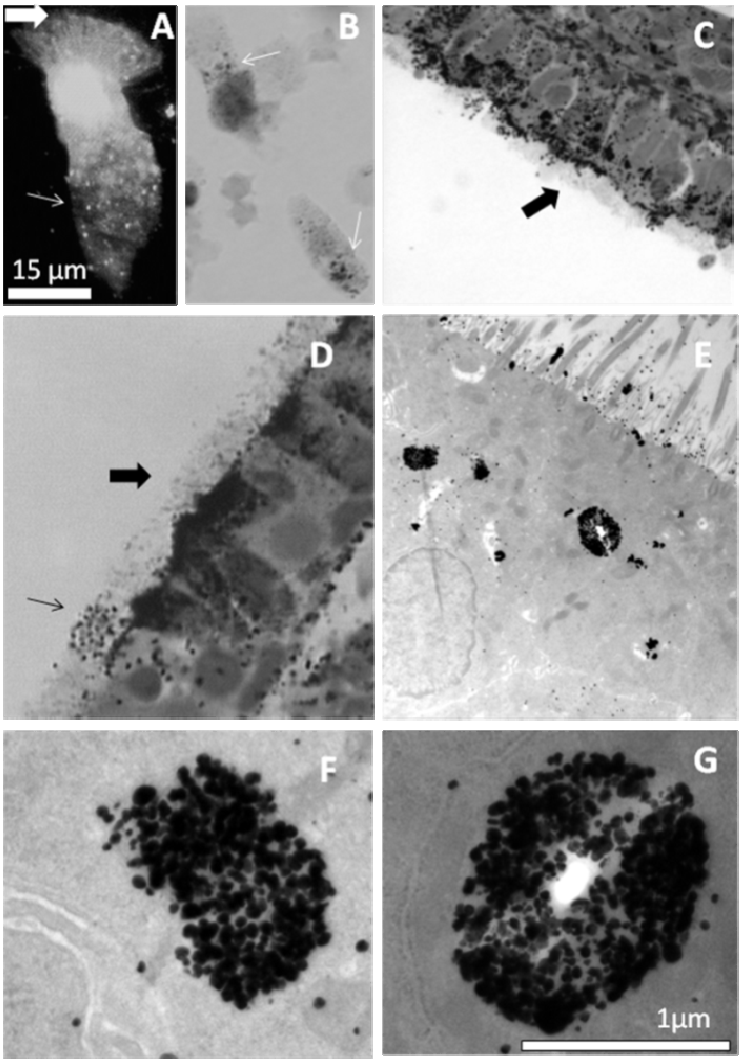
Zinquin fluorescence and autometallography of metal ions in apical cytoplasm of airway epithelium (AE) (**A**) Labeling of labile Zn by Zinquin in a typical human ciliated bronchial epithelial cell. Note the intense fluorescence in the apical cytoplasm, fluorescence in cilia (thick arrow) and vesicular-like fluorescence extending into the basal region of the cell (thin arrow); (**B**) *In vitro* silver enhanced ZnSe nanocrystals (thin arrows) in the apical cytoplasm of pig tracheal epithelial cells; (**C**-**G**) *In vivo* Se-AMG-stained rat tracheal epithelium. Note the labeling of Zn ions in the cytoplasm below the cilia (thick arrow). In one of the AE cells (Panel D, thin arrow), Zn ions are largely absent from the cytoplasm, but seem to have moved out of the cell and onto the surfaces of the cilia. In panels E-G, typical transmission electron micrographs show clusters of zinc-selenium nanocrystals in the apical cytoplasm and around the cilia. Some clusters (e.g., panel G), show a structure that appears to be enclosed by a membrane and to contain a hollow core surrounded by metal ion.

For labeling of Zn, brushed human ciliated columnar bronchial epithelial cells were treated in suspension with the Zn fluorophore Zinquin and examined by fluorescence microscopy. We have preferred to use Zinquin in these experiments rather than the more commonly used FluoZin3, as in our hands, Zinquin gives sharper images with these airway epithelial cells. Others have shown that Zinquin and FluoZin3 label different Zn-containing compartments in islet beta cells, granules and cytosol, respectively [33]. As previously shown [21], Zinquin stained AE cells in a vesicular manner throughout the cytoplasm but with a very high intensity in the ciliated, apical portion of the cell (thick arrow, typical shown in (Figure 1A). Close examination of the apical cytoplasm shows that the fluorescence was largely within vesicles as well as in the cilia. Some vesicles (thin arrow in Figure 1A) were also seen towards the basal end of the cell. Fluorescence was completely quenched by the specific, membrane-permeable Zn chelator TPEN (not shown). 

A vesicular localization of Zn ions was confirmed by Se-AMG. Figure 1B shows Zn-Se nanocrystals (thin arrows), in the apical cytoplasm of pig tracheal epithelial cells after exposure of the isolated cells to sodium selenide *in vitro*. Next, we introduced sodium selenide into the trachea of anaesthetized rats, followed by fixative. Bright field images show intense labeling of metal ions in the apical cytoplasm (thick arrows in Figure 1C,D). Interestingly, occasional cells showed staining restricted to only the cilia (thin arrow in Figure 1D), giving the impression that the cell had secreted its Zn ions across the apical membrane. Electron micrographs confirmed the strong labeling in the apical cytoplasm to be located both in vesicles and in the cytoplasm and on the surface of the lower part of the cilia (Figure 1E-G). 

### 3.2. Expression of Vesicular Zn Transporter ZnT4 by qPCR in Human AEC

**Figure 2:**
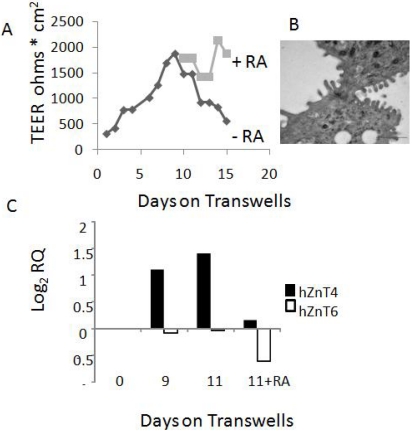
ZnT4 in air-liquid interface cultures of normal human bronchial/tracheal epithelial cells (NHBE). (**A**) Changes in transepithelial resistance (Trans-Epithelial Electrical Resistance (TEER), ohms × cm^2^) plotted against days in culture on transwell filters (see methods). Note the steep increase in TEER up to day 9 of culture and loss of TEER after day 9 in the absence, but not presence, of retinoic acid (RA); (**B**) Transmission electron micrograph showing a typical tight junction formed in cultures exposed to RA; (**C**) ZnT4 mRNA relative to hypoxanthine phosphoribosyltransferase (HPRT) increased during culture on filters but was lost following addition of RA. For comparison, ZnT6 did not increase during culture.

Our previous studies in whole mouse lung showed that ZnT4 is expressed at the mRNA level, along with a number of other ZIP and ZnT Zn transporters [25]. To confirm that ZnT4 is also expressed at the mRNA level in human AEC, we used real time qPCR initially on NHBE cells that had been seeded on filters in submerged cultures and after a few days converted to air-liquid interface (ALI) cultures to mimic airways environment [34]. ALI culture caused a marked increase of trans-epithelial electrical resistance (TEER), peaking at day 9 (Figure 2A); this was likely to be due to formation of tight junctions, an important feature of functional AE. Cultures beyond day 9 resulted in a dramatic decline in TEER but this decline could be reversed by addition of retinoic acid (RA) to the cultures (Figure 2A). Electron microscopy studies confirmed that these culture conditions promoted the formation of morphologically recognizable tight junctions (example shown in Figure 2B). Levels of ZnT4 mRNA increased almost 3-fold during ALI culture of NHBE cells on air-liquid interface, peaking at days 9-12 (Figure 2C). Addition of RA at day 11 resulted in a marked decrease in ZnT4 mRNA (Figure 2C). For comparison, we also studied expression of ZnT6 that remained stable in expression throughout the experiment (Figure 2C). Other data showed no change in expression of ZIPs 3, 7 and 8 during the differentiation, while ZIPs 4, 6 and 14 decreased (data not shown). 

**Figure 3:**
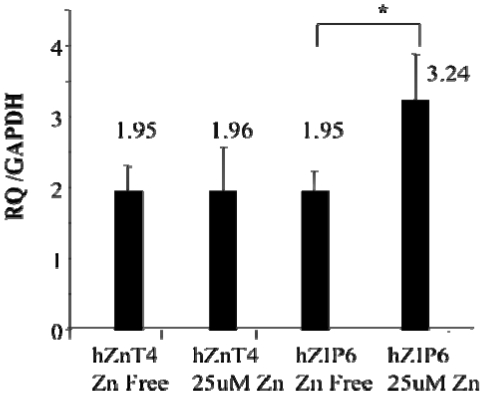
ZnT4 mRNA expression in human nasal AE was unaffected by extracellular Zn. ZnT4, unlike ZIP6, did not respond to an increase in extracellular Zn concentration (to 25 µM).

To determine whether primary human AE also expresses ZnT4 mRNA, human nasal AEC were obtained from three donors by nasal brushing and ZnT4 mRNA was assayed by real time qPCR. ZIP6 was also determined for comparison since it was previously shown to be up-regulated by exogenous zinc in the culture medium [35]. All three samples showed abundant expression of both transporters. Next, we determined whether levels of ZnT4 and ZIP6 were influenced by extracellular Zn. Human nasal AEC were cultured overnight in either Zn-depleted medium or Zn-depleted medium re-supplemented with 25 µM Zn sulphate. Figure 3 shows no change in ZnT4 mRNA levels. By contrast, there was a 1.7-fold increase in ZIP6 mRNA level in the Zn-supplemented cultures. 

### 3.3. Localization of Vesicular Zn Transporter ZnT4 in Primary Human AEC by Immunofluorescence

**Figure 4:**
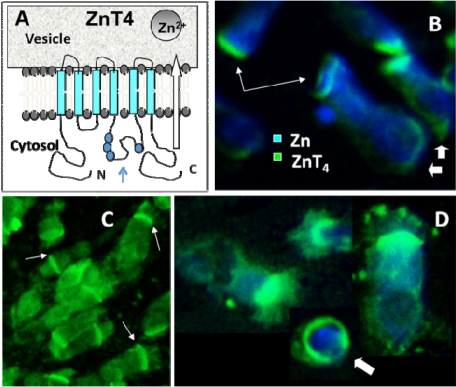
Localization of vesicular Zn transporter ZnT4 in primary human airway epithelial cells (AEC) by immunofluorescence (**A**) ZnT4 with 6 putative trans-membrane domains penetrates the membranes of cytoplasmic endosomes, vesicles and secretory granules with both *N*- and *C*-terminal ends and the histidine-rich loop, thought to bind cytosolic Zn, protruding into the cytosol; the large arrow shows the presumed direction of Zn movement out of the cytosol and into the vesicle; (**B**-**D**) Immunofluorescence labeling of ZnT4 (green) in human bronchial epithelial cells showing a tight band of fluorescence in the far apical cytoplasm and in the ciliaas well as occasional staining of basolateral membrane (B and C) and of the entire membrane in a non-columnar cell (arrow in D). Blue labeling in panels B and D depicts Zinquin fluorescence of dual-labeled human bronchial epithelial cells. The more diffuse blue staining of Zn ions is due to effects of fixative on subsequent Zinquin staining. Merged confocal images are shown. Thin arrows in B and C point to the apical staining while thick arrows in B point to the basal ends.

A schematic representation of ZnT4 protein membrane topology is shown in (Figure 4A). The protein possesses 6 putative trans-membrane domains (light blue) and resides in the membranes of intracellular vesicles and other organelles [22,27]. Thin arrow in (Figure 4A) denotes the putative Zn-binding, histidine-rich loop protruding into the cytoplasm. The intracellular distribution of ZnT4 protein was studied in primary human AE cells (both nasal and bronchial), using a specific polyclonal antibody previously described [22]. Immunofluorescence studies showed a predominantly apical cytoplasmic distribution of ZnT4 protein in freshly isolated human airway epithelial cells (Figure 4B-D), similar to the distribution of labile Zn (Figure 1A). The apical ZnT4 fluorescence was predominantly in the region just below the cilia, although, in some cells, fluorescence was observed in the cilia, themselves (Figure 4D). Some cells also had a rim of fluorescence at the basolateral membrane (e.g., Figure 4B, thick arrows), occasionally extending around the entire plasma membrane, especially in rounded up cells (Figure 4D). Secondary antibody alone also did not result in significant fluorescence (not shown). In singly-labeled cells, the fluorescence intensities for Zn and ZnT4 were quantified by image analysis within different subcellular compartments (see Methods). The order from most intense staining to least intense for ZnT4 was apical, perinuclear, nuclear, membrane, cilia and basal. For zinc the order was apical, cilia, perinuclear, nuclear, membrane then basal. 

Next, we attempted to study the co-localization of ZnT4 and Zn, using dual-labeled cells (anti-ZnT4 and Zinquin, (Figure 4B,D). There was considerable overlap of Zn (blue) and ZnT4 (green) fluorescence; however, the Zinquin fluorescence was often much more diffuse and lower in intensity than in cells stained with Zinquin alone. This was an artifact due to the effect of the fixative/permeabilization technique (required for the immunofluorescence assay) on the labile Zn compartment.

### 3.4. Comparisons between Nasal and Bronchial Human AEC

To determine whether the staining patterns for Zn and ZnT4 in nasal AEC were similar to those in lung AEC, staining patterns and fluorescence intensities for ZnT4 and Zn were compared for AEC derived by bronchial brushing (*n* = 5, obtained during routine bronchoscopies) with those for AEC derived by nasal brushing (*n* = 5). The patterns of Zn and ZnT4 distribution were similar in the two types of cells (not shown) and there were reasonably good correlations between levels of ZnT4 in nasal *versus* bronchial AEC (*r*^2^ = 0.76) and between levels of labile Zn in nasal *versus* bronchial AEC (*r*^2^ = 0.57). There was, however, a tendency for higher Zn values in the bronchus compared to the nose. The opposite was found with ZnT4 where there was significantly higher intensity (*P* = 0.008) in the nasal samples compared with the bronchial. 

### 3.5. Down-Regulation of ZnT4 Protein in AE of Mice with Allergic Airway Inflammation

**Figure 5:**
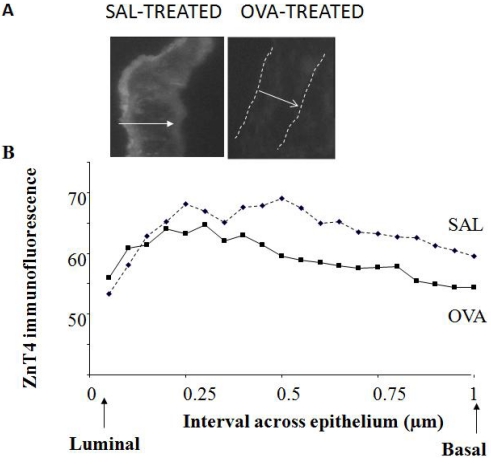
Loss of ZnT4 staining in AE of ovalbumin (OVA)-treated mice with airway inflammation (**A**) Figure shows ZnT4 immunofluorescence labeling in AE of a typical sham-treated control Balb/C mouse (left) and OVA-treated Balb/C mouse (right). In the latter photomicrograph, the dashed lines indicate the orientation of the epithelium. The arrow indicates the width of the epithelium from luminal (tail of arrow) to basal (head of arrow). Note the stronger ZnT4 fluorescence at the luminal (apical) end in the control section and the loss of fluorescence in the OVA-treated mouse AE; (**B**) Figure shows the loss of ZnT4 fluorescence averaged over a number of mice. Mean fluorescence intensity per interval across the epithelium is shown, from luminal end (left) to basal end (right). Means were from multiple images (5-10 per mouse) for a total of 10 SAL and 11 OVA mice. There was a significant loss of ZnT4 staining in the OVA-treated mice (*P* < 0.005), preferentially in the basal compartment.

To confirm the presence of ZnT4 transporter protein in murine AE and its down-regulation during airway inflammation, ZnT4 protein was labeled by indirect immunofluorescence in cryo-sections of lung airways from control (SAL-treated) or airway-inflamed (OVA-treated) mice. (Figure 5A) shows typical images for ZnT4 in AE of SAL and OVA mice and (Figure 5B) shows the intensity across the epithelium from luminal (left) to basal (right) ends, averaged over 5-10 images for 10 SAL and 11 OVA mice (see Methods for detail). There was negligible fluorescence in AE stained with secondary antibody alone (not shown). For the entire epithelium, the mean ± SD ZnT4 fluorescence (arbitrary fluorescence units) for the SAL group was 63.8 ± 3.9 compared to the OVA group 59.2 ± 3.3. The difference was highly significant (*P* = 0.0002, Student *t* test, 2-tailed). For the apical side of the epithelium (intervals 1-7), the mean ± SD ZnT4 fluorescence (arbitrary fluorescence units) for the SAL group was 62.8 ± 5.3 compared to the OVA group 61.8 ± 2.9. The difference was not significant (*P* = 0.67, Student *t* test, 2-tailed). For the basal side of the epithelium (intervals 8-20), the mean ± SD ZnT4 fluorescence (arbitrary fluorescence units) for the SAL group was 64.3 ± 3.1 compared to the OVA group 57.8 ± 2.6. The difference was highly significant (*P* = 0.000006, Student *t* test, 2-tailed).

In control mice, the expression of immuno-reactive ZnT4 was found throughout the AE but most pronounced at the luminal end. There was a significant and substantial decrease in the ZnT4 expression of the OVA mice especially towards the basal end but no significant decrease at the apical end.

## 4. Discussion

This study provides three major findings and an exciting new hypothesis: (1) EM tracing of Zn ions in clusters of vesicle-like organelles in the apical cytoplasm of AE and on the surface of the lower third of the cilia; (2) Immunohistochemical tracing of vesicular Zn transporter ZnT4 predominantly localized to the Zn-enriched apical cytoplasm but also at the basal end, suggesting its contribution to Zn homeostasis in this tissue; and (3) a parallel loss of ZnT4 protein and Zn ions in AE of mice with airway inflammation. Several models involving different animal species were used in this study, because of the specific techniques being used in the different experiments. Findings with the different species support the generality of the conclusions. We believe that these findings have important implications for the mechanisms of altered Zn homeostasis and Zn deficiency in chronic airway inflammatory disease. 

We have previously shown striking losses of both airway epithelial Zn [13] and whole lung ZnT4 mRNA [25] in mice with allergic airway inflammation. In this study we have confirmed the ZnT4 gene expression in human AEC at the protein level. We have also found that the ZnT4 mRNA expression in AE cells was not affected by adding Zn ions to the medium while the ZIP6 was up-regulated. ZIP proteins are thought to be involved in cellular Zn uptake [24]. ZIP6 is one candidate for the plasma membrane Zn transporter that brings Zn ions into the AE cells, although other ZIP transporters are expressed in AE and require further study. Of particular interest are the recent elegant studies by Daren Knoell’s group [36,37] showing a specific role for another ZIP transporter ZIP8 in mediating uptake of Zn by human airway epithelial cells (primary and BEAS-2B) in response to a pro-inflammatory stimulus TNFα and a role for ZIP8 in maintaining Zn levels in these cells required for epithelial cell monolayer integrity and cell survival as well as roles for ZIP8 in sepsis. Their studies showed that some of the imported zinc ended up in cytoplasmic vesicles in the airway epithelial cells [35]. It may be that ZIP8 and ZnT4 act in concert to maintain intracellular levels of Zn in airway epithelium, mediating plasma membrane uptake followed by vesicular localization of Zn, respectively. It is not clear whether AEC derive the bulk of their Zn from sub-epithelial capillaries or can absorb Zn across their apical membranes from epithelial secretions or plasma exudates. A study of the distribution of ZIP transporters through the basal-apical axis of AE cells followed by functional analysis needs to be carried out to answer this question. 

The experiments with the Se-AMG technique confirm the Zinquin findings of high levels of loosely bound or free Zn ions in the apical cytoplasm of AEC. Of particular interest from the EM studies was the presence of Zn-selenium nanoparticles in the apical vesicular-like structures. Similar apical Zn-rich structures have previously been reported in the prostate epithelium where they mediate secretion of Zn into the prostatic fluid and subsequently into ejaculated spermatozoa [38,39], in the synaptic vesicles of Zn-enriched (ZEN) neurons of the CNS and in the salivary glands of rats and multiple other secretory cells including Paneth cells and mast cells (22). The Zinquin fluorescence and ZnT4 immunofluorescence studies in both isolated human AEC and mouse AE cryosections suggest a prominent role for ZnT4 in redistribution of Zn within AE. The “starry sky” staining pattern of labile Zn in the apical cytoplasm and the coinciding patterns of labile Zn and ZnT4 indicate a sequestering role for ZnT4 in the membranes of cytoplasmic Zn vesicles (zincosomes) on the apical side of polarized epithelial cells. The term “zincosome” refers to vesicular pools of labile Zn in cells [40,41]. Free or loosely bound Zn ions seem to be involved in apoptosis when present in the cytoplasm under certain circumstances. They have been found to bind to and inactivate essential sulphydryls at the active sites of cytoplasmic enzymes [5]; however in most cases loosely bound or free Zn ions are beneficial and supportive (e.g., in wound healing and storage of proteins in vesicles such as insulin in the beta-cells of the pancreas).

The observation that ZnT4 is partially lost from AE during murine airway inflammation supports our previous findings that inflammation causes ZnT4 mRNA down-regulation [30] and loss of labile Zn staining [17]. There is evidence that some asthmatics have low systemic levels of Zn (serum/plasma and hair Zn). However, whether this is equivalent to clinical Zn deficiency has yet to be determined (discussed in [18]). Plasma Zn constitutes less than 1% of total body Zn [42] and estimations based on blood may therefore not be representative for the level of free or loosely bound levels of Zn in airway tissue. We have recently shown that the levels of Zn ions in induced sputum are decreased in older patients with asthma [43]. Measuring Zn levels in bronchial brushings presents a technical problem in that these brushings are not routinely performed in asthmatic subjects. Nasal epithelial cells, however, are more easily obtained by nasal brushing. Several studies have shown that nasal epithelial cells resemble bronchial epithelial cells from the same subject in a number of properties [44,45]. Here we have shown reasonably good correlations between bronchial and nasal Zn staining from the same subject, as well as between bronchial and nasal ZnT4 staining. 

**Figure 6:**
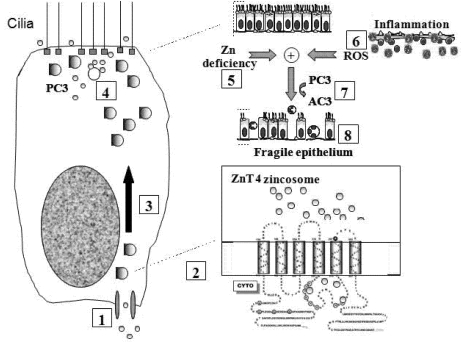
Model for role of ZnT4 in AE Zn homeostasis and airway inflammation: Step **1**: Zn is taken up across the basolateral plasma membrane of AEC from sub-epithelial capillaries via ZIP6 or other ZIP transporter(s); Step **2**: Zn is incorporated into vesicles with the aid of ZnT_4_ (see inset); Step **3**: These vesicles translocate to the apical cytoplasm; Step **4**: Apical cellular Zn may protect the cytoplasmic mucociliary apparatus (e.g., tubulin and basal bodies) from damage by oxidants and other toxins that would otherwise trigger pro-caspase-3 (PC3) activation. Vesicular Zn may also be secreted across the apical plasma membrane into the epithelial lining fluid, cilia and mucin; Step **5**: Apical cellular Zn is depleted in chronic inflammatory airway disease. Mechanisms may include abnormalities in ZnT_4_, hyper-secretion of Zn or excessive loss of Zn by luminal shedding of dying AEC or exudation of inflammatory cells; Step **6**: Depletion of Zn renders AEC vulnerable to reactive oxygen species (ROS) released from inflammatory cells or mitochondria; Step **7**: This leads to premature activation of PC3 to active caspase-3 (AC3) and downstream events in apoptosis. Zn depletion also directly facilitates PC3 activation because this enzyme is inhibited by binding of Zn to an essential sulphydryl group [5]; Step **8**: The altered epithelium has increased apoptosis and epithelial sloughing, which contribute to the ongoing inflammation.

Figure 6 shows a model incorporating the specific role of ZnT4 in airway Zn homeostasis, the normal functions of apical vesicle Zn in AE and the abnormalities that accompany or promote airway inflammatory disease. We propose that Zn is transported into the AE cells by way of one or more ZIP transporters and stored in the apical vesicles by ZnT4. The finding that Zn repletion did not increase the transcription of ZnT4, but increased ZIP6 transcription, argues against a role for ZnT4 in the plasma membrane uptake of Zn. However, as our immunofluorescence studies suggested that some cells do appear to have plasma membrane ZnT4, a role also in Zn uptake cannot be entirely ruled out. The presence of a significant pool of ZnT4 at the basal end of the cells is consistent with the hypothesis that ZnT4 receives Zn as it is imported across the basal plasma membrane. If this is the case, the preferential depletion of the basal ZnT4 in the OVA-treated mice would be consistent with an interruption of the uptake and packaging of Zn during airway inflammation. The primary role of ZnT4 appears to be incorporation of cytosolic Zn ions into vesicles for transport to the apical region. Apical cytoplasmic Zn-regulated proteins include Cu/Zn superoxide dismutase, a major anti-oxidant in AE, and procaspase-3, a pro-apoptotic effector [21]. The presence of Zn ions in these enzymes is consistent with cytoprotective anti-oxidant and anti-apoptotic roles for labile Zn in AE [5]. 

The protective role of Zn ions might also explain the Zn-selenium nanoparticles found adjacent to the bases of the cilia. These microtubule-rich organelles are vulnerable to oxidative stress [46] and Zn is known to protect tubulin from oxidation in cell-free extracts [47]. Impairment of the beating of cilia in Zn depleted AEC would be consistent with our previous studies showing substantial oxidative damage in the apical and ciliary membranes of Zn depleted human AEC [21]. Such damage might lead to paralysis of the cilia and, eventually, epithelial cell death. These processes would likely be facilitated in airway inflammatory disease where there is both Zn depletion in AE and oxidant release from infiltrating eosinophils and neutrophils. The observation of ZnT4 near the base of the cilia and the images showing occasional cells with Zn-depleted cytoplasm and Zn-rich cilia (e.g., Figure 1D) suggest that some of the vesicular Zn is destined for secretion across the apical membrane and into the epithelial lining fluid. A role for ZnT4 in secretion of AE Zn is not unlikely, as it is involved also in Zn secretion into breast milk [32,33,37].

## 5. Conclusions

In summary, we have now characterized the distribution of a major Zn transporter ZnT4 in human airway epithelial cells and compared it at two sites: the nasal and bronchial mucosa. The subcellular distribution of ZnT4 supports the notion that Zn ions have significant roles in normal function of the mammalian airway epithelium, while the loss of ZnT4 in a murine asthma model implicates it as a factor in the mechanisms leading to aberrant Zn homeostasis, epithelial vulnerability and possibly ciliary dysfunction in airway inflammatory disease. However, further evidence including targeted up- or down-regulation of ZnT4 is required to prove a functional role for ZnT4 in maintaining zinc homeostasis within the airway epithelium while studies with other proinflammatory mediators (e.g., TNFα) will indicate the generality of the findings in other models of inflammation. 
